# Polyunsaturated fatty acid balance modulates microglial state in a murine model of oxygen-induced neovascularization

**DOI:** 10.21203/rs.3.rs-7491382/v1

**Published:** 2025-09-23

**Authors:** Esther S. Kim, Meng-Chin Lin, Cheng-Hsiang Lu, David Casero, Brian Aguirre, Joanne Brown, Olawande Olagoke, Camilia R. Martin, Madhuri Wadehra, Kara L. Calkins, Alison Chu

**Affiliations:** 1 Department of Pediatrics, Olive View-UCLA Medical Center, 14445 Olive View Drive, Sylmar, CA, 91342, USA; 2 Division of Neonatology and Developmental Biology, Department of Pediatrics, David Geffen School of Medicine, University of California-Los Angeles, Los Angeles, CA 90095; 3 F. Widjaja Inflammatory Bowel Dise ase Institute, Cedars Sinai Medical Center, Los Angeles, CA 90048; 4 Department of Pathology Lab Medicine, David Geffen School of Medicine, University of California-Los Angeles, Los Angeles, CA 90095; 5 Department of Gastroenterology, Beth Israel Deaconess Medical Center, Boston, MA 02215; 6 Division of Neonatology, Department of Pediatrics, Weill Cornell Medicine, New York, NY 10065; 7 Jonsson Comprehensive Cancer Center, UCLA, Los Angeles, CA 90095

**Keywords:** retina, retinopathy of prematurity, oxygen-induced retinopathy, polyunsaturated fatty acids, angiogenesis, hypoxia, microglia

## Abstract

The Oxygen-Induced Retinopathy (OIR) model is a widely used research tool to study pathological retinal neoangiogenesis observed in the development of diseases like retinopathy of prematurity (ROP) and proliferative diabetic retinopathy. While many factors are known to modulate susceptibility to OIR development, less is understood about how nutritional factors such as dietary fatty acids affect disease progression. The retina is enriched in polyunsaturated fatty acids (PUFAs) which are indispensable for normal vision, and recent work has shown that dietary supplementation of ω−6-and ω−3-polyunsaturated fatty acids (PUFAs) can provide a protective role against the pathological neovascularization observed in ROP. In the current study, we interrogated the effects of endogenous ω−3-PUFA enrichment using transgenic *fat-1* mice which converts ω−6-PUFAs to ω−3-PUFAs. These animals develop features of ROP but show attenuation in retinopathy development. Using a combination of immunofluorescence and whole retinal RNA sequencing, genes associated with angiogenesis, inflammation, and microglial activation were upregulated in WT OIR vs. WT RA (room air), with little or no change found in *fat-1* OIR vs. *fat-1* RA. In addition, *Fat-1* mice demonstrated differential enrichment of microglial subtypes in response to OIR. This finding suggests that decreased ω−6:ω−3 protects against neovascularization by attenuating hyperoxia-induced microglial activation.

## INTRODUCTION

Retinopathy of prematurity (ROP) affects approximately 1 in 3–4 extremely premature infants.^1,2^ The incidence of ROP has increased with improved survival of preterm infants.^3^ Severe ROP is associated with an increased risk of neurological disability^4,5^ and can culminate in retinal detachment and blindness. Laser ablation and anti-vascular endothelial growth factor (VEGF) injections are the only treatments for ROP. However, these treatments are invasive, treat the late phase of ROP, and are associated with increased risk of myopia, cataracts, glaucoma, and retinal thinning.^6^ While limiting oxygen exposure mitigates ROP risk, clinicians must balance this risk with adequate oxygen support to sustain tissue oxygenation. Currently, there are no other widely accepted preventative or treatment strategies for ROP.^7^

**Figure 1:**
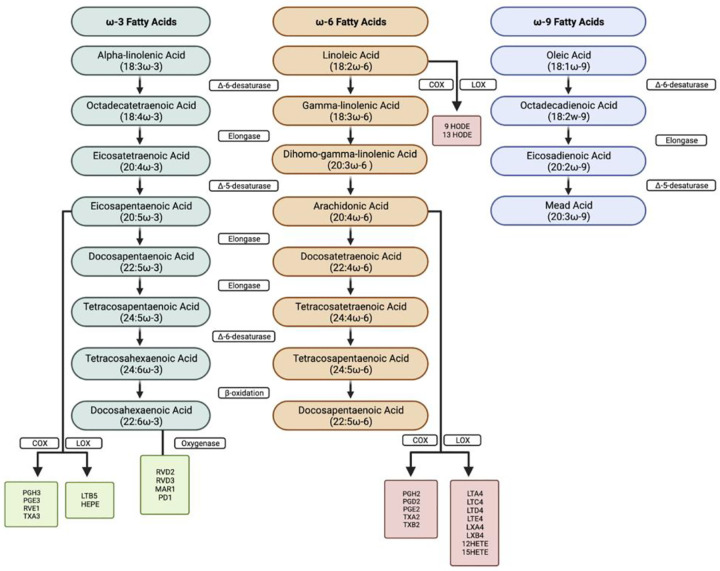
Metabolism of polyunsaturated fatty acids and their downstream products. COX, cyclooxygenase; LOX, lipoxygenase; PGH2, prostaglandin H2; RVE1,resolvin E1; TXA2, thromboxane A2; HEPE, hydroxy-eicosapentaenoic acid; RVD2, resolvin D2; RVD3, resolvin D3; MAR1, maresin 1; PD1, protectin 1; PGH2, prostaglandin H2; PGD2, prostaglandin D2; PGE2, prostaglandin E2; TXA2, thromboxane A2; TXB2, thromboxane B2; LTA4, leukotriene A4; LTC4, leukotriene C4; LTD4, leukotriene D4; LTE4, leukotriene E4; LXA4, lipoxin A4; LXB4, lipoxin B4; HETE, hydroxy eicosatetraenoic acid; HODE, Hydroxy-octadecadienoic acid.

The etiology of ROP is multi-factorial; ROP is spurred on by complex interactions that involve inflammation, oxidative stress, pathological angiogenesis, and altered immune cell recruitment and activation.^7–12^ The polyunsaturated fatty acids (PUFAs), arachidonic acid (ARA) and docosahexaenoic acid (DHA), representative of ω−6 and ω−3 PUFAs, respectively, play an important role in retinal development and have been implicated in the development and progression of ROP.^8,9,10^ ARA and DHA are the most abundant PUFAs in the human retina.^11^ PUFAs are structural components of the lipid membrane, alter gene expression by activating nuclear transcription factors,^12–15^ and serve as substrates for bioactive lipid molecules, also known as oxylipins and specialized pro-resolving mediators (SPMs) (Figure 1). These molecules regulate diverse processes such as inflammation, oxidative stress, and angiogenesis.^13,14^

In preterm infants, circulating PUFA levels decline after birth despite parenteral and enteral nutrition.^15,16^ Low ARA and DHA status have been associated with ROP.^8,17^ In a multicenter, randomized controlled study of preterm infants at risk for ROP, enteral ARA and DHA supplementation was associated with a 50% decrease in severe ROP (RR 0.50, 95% CI 0.28–0.91, p=0.02).^9^ In a recent meta-analysis sponsored by the American Society for Parenteral and Enteral Nutrition, fish oil-containing intravenous lipid emulsions, a source of DHA and ARA, were associated with a decrease in the incidence of ROP stage 3 or greater compared to the standard soybean-based lipid emulsion, which is devoid of ARA and DHA (RD; −0.04, 95% CI, −0.08 to −0.01, p=0.02).^18^ However, the underlying mechanism of how PUFAs protect against retinal injury in preterm infants is not completely understood.

Mouse models of oxygen-induced retinopathy (OIR) mimic the phases of pathological vascularization in ROP and are used to study retinal angiogenesis. OIR consists of a vaso-obliteration phase fueled by hyperoxia exposure, followed by aberrant neovascularization induced by relative local hypoxia.^19,20^ Mouse models of OIR provide experimental proof that PUFAs play a role in ocular disorders.^21–23^ Using *fat-1* transgenic mice, Connor *et al*. showed that manipulation of retinal ω−6: ω−3 in mice could reduce the pathophysiological effects of hyperoxia exposure, independent of diet manipulation.^21^ *Fat-1* mice encode a functional desaturase enzyme that converts ω−6 PUFAs to ω−3 PUFAs.^24,^ 20 As a result, *fat-1* mice demonstrate a shift towards an ω−3 dominant PUFA profile in the retina, altering the ω−6:ω−3 ratio to 0.45 from 1.22 in wild type (WT) mice. The experiments by Connor *et al*. provided evidence that a relative increase in ω−3 PUFAs and decrease in ω−6 PUFAs protected against pathologic neovascularization in OIR. These experiments also demonstrated suppression of tumor necrosis factor-*α* expression and upregulation of DHA-derived SPMs (neuroprotectin D1, resolvin D1, and resolvin E1 (RVE1) from retinal microglia, the resident macrophages in the neuroretina.^21^

Retinal microglia protect against vascular damage by responding to inflammation and injury. In a study by Liu *et al*., single cell transcriptome analysis was used to map various microglia populations using a mouse model of OIR.^25^ The investigators identified highly proliferative and hypermetabolic subsets of microglia in mice exposed to OIR. However, the effects of retinal PUFAs on the inflammatory and immunogenic milieu in OIR is still relatively unknown. Thus, we sought to better characterize how microglia are altered by the retinal PUFA balance. In this paper, we utilized RNA-sequencing and microscopy studies to better understand how retinal microglia and changes in PUFA balance influence vascular health. We hypothesized that a reduced retinal ω−6:ω−3 ratio would attenuate OIR by altering the balance of microglial states which affect hypoxia-induced inflammation and angiogenesis.

## METHODS

### Animal Model

This study was approved by the Animal Research Committee at the University of California, Los Angeles (UCLA) and was in accordance with the National Institutes of Health guidelines. This study was reported in accordance with ARRIVE guidelines. Three transgenic *fat-1* mouse breeding pairs were gifted from the laboratory of Dr. Camilia Martin (Weill Cornell Medicine). This transgenic mouse has the *fat-1* gene of the C. elegans roundworm on a C57BL/6 background. The *fat-1* gene encodes an ω*−*3 fatty-acid desaturase enzyme that converts ω*−*6 PUFAs to ω−3 PUFAs.^24^

The UCLA Department of Laboratory Animal Medicine (DLAM) oversaw the colony breeding and maintenance. Genotyping was performed on tail snips to confirm *fat-1* mice, using primers: 5’- CTG CAC CAC GCC TTC ACC AAC C-3′ (forward) and 5′-ACA CAG CAG CAG ATT CCA GAG ATT-3′ (reverse) made through Integrated DNA Technologies (Coralville, IA, USA). Wild type (WT) C57BL/6 mice (Charles River Laboratories) were used as a control group and had *ad libitum* access to standard rodent chow diet (Pico Lab Rodent Diet 20, cat# 5053, Lab Diet, St. Louis, MO). *Fat-1* mice had *ad libitum* access to Mod TestDiet 58B0 with 10% corn oil (cat#5T9W, Lab Diet, St. Louis, MO) to provide a ω−6 dietary substrate.^21^ The diets and their fat content are described in Supplemental Table 1. *Fat-1* and WT mice were maintained in 12:12 hour light-dark cycles.

### Experimental Model of OIR

**Figure 2:**
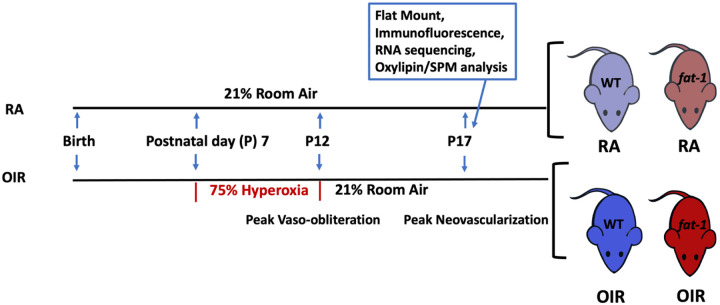
Murine model of oxygen induced retinopathy (OIR) and experimental design. OIR mimics the human vascular attention and vaso-proliferative phase of retinopathy by systemic exposure to hyperoxia. WT (blue) and *fat-1* (red) mice were kept in room air as controls. WT and *fat-1* mice were exposed to high oxygen (75%) from postnatal day 7 to 12 (OIR). P12 represents peak retinal vaso-obliteration and P17 represents peak neovascularization in OIR. Samples were collected at P17 to examine peak disease state.

Mouse models of OIR were generated as previously published.^19,20^ Once pups were delivered, they were set at postnatal day (P) 0.5 on the morning of discovery. *Fat-1* and WT mothers were maintained on their respective diets during gestation and suckling. Mothers and litters were randomly assigned to conditions of OIR or room air (RA). Mothers and litters assigned to the OIR group were placed into a hyperoxia chamber (75% oxygen) on P7 until P12. The hyperoxia chamber (Biospherix Proox model 360, Parish, NY, USA) conditions such as temperature, humidity and mouse health were monitored and checked twice a day while animals were in the chamber. Mice were removed from the hyperoxia chamber on P12 and placed back into RA (21% oxygen). When mothers were in poor health after the stress of the hyperoxia chamber, pups were fostered with a genotype-matched nursing mother. Samples were collected at P17, which represents peak neovascularization (Figure 2).

At the time of dissection, mice were anesthetized with isoflurane. Retinal tissues were dissected and processed for downstream application. Some samples were flash frozen in liquid nitrogen and stored at −80°C for processing. Tissues dissected for paraffin embedding were placed in formalin and then stored in 70% ethanol. Samples were collected from 3–4 litters at P17 for each condition. Approximately 4–9 mice representing at least 3 litters were analyzed per condition and genotype confirmed, leading to the generation of four groups: WT mice in RA (WT RA), WT mice in OIR conditions (WT OIR), *fat-1* mice in RA (*fat-1* RA), and *fat-1* mice in OIR conditions (*fat-1* OIR) (Figure 2).

### Whole Mount Imaging

After enucleation, retinal samples were prepared for whole mount images as previously published.^26^ Briefly, eyes were dissected using a surgical microscope (Leica S6D) and fixed in 4% formaldehyde for 1.5 hours at room temperature. Dissected retina cups were washed in phosphate buffered saline (PBS) and placed in a blocking buffer (20% FBS, 2% goat serum, 0.05% BSA, 1% TritonX-100 in PBS). Retinas were stained with Alexa594-isolectin GS-IB4 (Invitrogen, Carlsbad, CA) and stored overnight at 4°C in diluent buffer. The next day, retinas were washed with PBS and then mounted onto slides by making incisions at each corner to yield four quadrants. The retinas were mounted with ProLong (Invitrogen, Carlsbad, CA). The flat mounted slides were imaged using an AxioCam CCD digital camera mounted to an inverted epifluorescence microscope (AxioVert 135; Carl Zeiss).

### Quantification of Vaso-obliteration and Neo-vascularization in OIR at P17

Whole mount images were uploaded and processed in Image J. As previously published, images from P17 were quantified for areas of vaso-obliteration and neovascularization.^27^ A blinded scorer quantified areas of vaso-obliteration and neovascularization. The polygonal Lasso tool was used to quantify the total retinal area and area of vaso-obliteration. The quantified number of pixels of vaso-obliteration was then calculated as a percentage of the whole retina. Then the magic wand tool was used to manually outline individual neovascular tufts and unorganized areas of leaky small vessels with a threshold set at 50. The quantified number of pixels of vascularization was then calculated as a percentage of the whole retina.

### Immunofluorescence staining

Eye globes from P17 mice were enucleated, fixed in formalin for 24 hours and then placed into 70% ethanol. Samples were processed for embedding by the UCLA Department of Pathology’s Translational Pathology Core Laboratory (TPCL). Globes were embedded in paraffin and cut into 4 μm sections. One slide was stained with hematoxylin and eosin. Paraffin-embedded retina sections were stained for microglia using anti-Iba1 rabbit monoclonal antibody (Cell signaling, cat #17198 Danvers, MA, USA) at a 1:100 dilution, and for endothelial cells using Lycopersicon esculentum (Tomato) Lectin (Vector Laboratories, cat #FL-1171) at a 1:850 dilution. Donkey anti-Rabbit 594 (Invitrogen, cat #SA5–10040 Carlsbad, CA, USA) was used for the secondary antibody at a 1:300 dilution. Prolong Gold Antifade Mountant with DAPI (Fisher Scientific, cat # P36935 Hampton, NH, USA) was used as the final step to mount slides and stain nuclei. Slides treated with secondary antibody only were used as controls.

Slides were then imaged at 40x magnification using an AxioCam CCD digital camera mounted to an inverted epifluorescence microscope (AxioVert 135; Carl Zeiss). Images were quantified blindly using Adobe Photoshop software (Version 23.3.1, San Jose, CA, USA). The scorer used the histogram command to score the mean intensity of the red channel by averaging three 100 by 200-pixel boxes for each slide at the region of interest.

### RNA Sequencing Libraries and Data Analysis

Retina samples were dissected from WT and *fat-1* mice in conditions of RA and OIR at P17 and flash frozen. The retinal tissue was suspended in lysis buffer and sonicated. Then the tissue was homogenized using the QiaShredder kit (Qiagen, cat# 79654, Valencia, CA). Once homogenized, the RNA was extracted using the Qiagen RNeasy Mini Kit (Qiagen, cat# 74104, Valencia, CA). Samples were then sent to the UCLA Technology Center for Genomics and Bioinformatics for processing. Samples were quantified and tested for RNA degradation and determined adequate for RNA sequencing. Libraries for RNA-Seq were prepared with the KAPA Stranded mRNA-Seq Kit. The workflow consisted of mRNA enrichment and fragmentation, first strand cDNA synthesis using random priming followed by second strand synthesis converting cDNA:RNA hybrid to double-stranded cDNA (ds-cDNA), and incorporation of dUTP into the second cDNA strand. cDNA generation was followed by end repair to generate blunt ends, A-tailing, adaptor ligation and PCR amplification. In one lane, different adaptors were used for multiplexing sample. Sequencing was performed on Illumina HiSeq 3000 for SE 1×65bp run.

Illumina’s SAV was used for data quality checking. Illumina Bcl2fastq v2.19.1.403 software was used for demultiplexing. The raw RNA sequencing data unique to this study have been deposited in the National Center for Biotechnology Information (NCBI) Short Read Archive (SRA) with accession code PRJNA1006021. Data from our previous study^26^ is available at NCBI’s Gene Expression Omnibus (GSE123945), and was re-analyzed and integrated to incorporate transcriptional changes for P17 WT mice.

Sequence reads were aligned to a genome index that included both genome sequence (GRCm39 mouse primary assembly) and the exon/intron structure of known mouse gene models (Gencode M31 comprehensive genome annotation) using STAR v2.7.10b.^28^ Alignment files were used to generate strand-specific gene counts. Independent filtering was applied to remove low count genes and only protein coding genes were considered for downstream analysis. Expression estimates are reported in units of transcripts per million (TPM) and were computed from the filtered counts matrix after correcting for gene mappable length and per-sample sequencing depth as before.^26^ Estimates of cell type proportions for all TPM-normalized samples were obtained using the Gene Expression Deconvolution Interactive Tool (GEDIT^29^) with signatures from the Tabula Muris Reference database.^30^ To gain more insight into the microglial enrichment and activation shifts induced by OIR, we performed Gene Set Variation Analysis^31^ to estimate enrichment scores on microglia gene signatures from the Mouse Body Atlas and recent high-resolution single-cell studies.^25,32–35^

Unless otherwise noted, count-based and variance-stabilized data (*vsd*)^36^ were used for all ordination, differential and clustering analysis and all figures. Principal component analysis (PCA) was performed using the *vsd* matrix in R.^37^ Differential expression analysis was performed with DESeq2.^36^ For pair-wise tests, genes were classified as regulated when Wald adjusted p < 0.05.

Functional analysis of differentially expressed genes was performed with Metascape^38^ and enrichment statistics are presented as hypergeometric adjusted p values. All plots were generated in R^37^ and Matlab (MATLAB, version release 2020b, The MathWorks, Inc, RRID:SCR_001622).

### Retinal Oxylipins and Special Resolving Mediators at P17

To explore the relative levels of ω−6 and ω−3 PUFA inflammatory products in OIR, we used retinal samples for select analysis of oxylipins and SPMs. Sample preparation and analysis were carried out as described previously.^39^ Retinal cup samples, ranging from 2–8.4 mg (wet weight), were homogenized in 50 μl of filtered 1X PBS. 5 μl of 20 pg/μl internal standard and 945 μl of methanol were added to the 50 μl of homogenized sample, vortexed for 5 seconds, allowed to sit in ice for 10 minutes and centrifuged at 15,000 g for 30 minutes at 4°C. Clear supernatant was transferred to a new tube and dried with a nitrogen evaporator (Organomation, USA) at 37° C. Samples were resuspended in 50 μl of water and methanol (1:1 mixture), vortexed for 5 seconds, then centrifuged at 15,000 g for 1 minute at 4° C. The supernatant was then transferred into amber vials for the oxylipin assay via liquid chromatography with tandem mass spectrometry (LC-MS/MS). The LC-MS/MS device was comprised of an Ultra-High-Performance Liquid chromatography (UHPLC) on the Kinetex^®^ LC column [2.6 μm Polar C18 100 Å 100 × 3.0 mm (Phenomenex, USA)], and a tandem mass spectrometry on the hybrid triple quadrupole (QTRAP 6500+) system with multi-component IonDrive^™^ technology (AB SCIEX LCC, USA). Data was acquired in the Analyst^®^ Software (version 1.7.2) based on a targeted scheduled multiple reaction monitoring-information dependent acquisition-enhanced product ion (sMRM-IDA-EPI) method and quantified in the SCIEX OS with parameters shown in Supplemental Table 2.

### Statistical Analysis

All statistical analysis was performed using GraphPad Prism (Version 6, GraphPad Software Inc., La Jolla, CA) except for RNA sequencing data. Kruskal-Wallis testing was used to compare more than two non-parametric data sets (flat mount vaso-obliteration and neovascularization, retinal oxylipins and SPMs). Post-hoc Dunn’s multiple comparison testing was used to compare two non-parametric data sets. These data are presented as median with interquartile range (25^th^ percentile, 75^th^ percentile) and p<0.05 was used for statistical significance. ANOVA one-way test followed by Bonferroni’s multiple comparisons test was used for microglial score and lectin score for immunofluorescence. These data are presented as mean (SEM) and p<0.05 was used for statistical significance. For each condition, 4–9 pups obtained from at least 3 litters were analyzed.

## RESULTS

### *Fat-1* mice demonstrate attenuation of pathologic neovascularization in OIR

**Figure 3:**
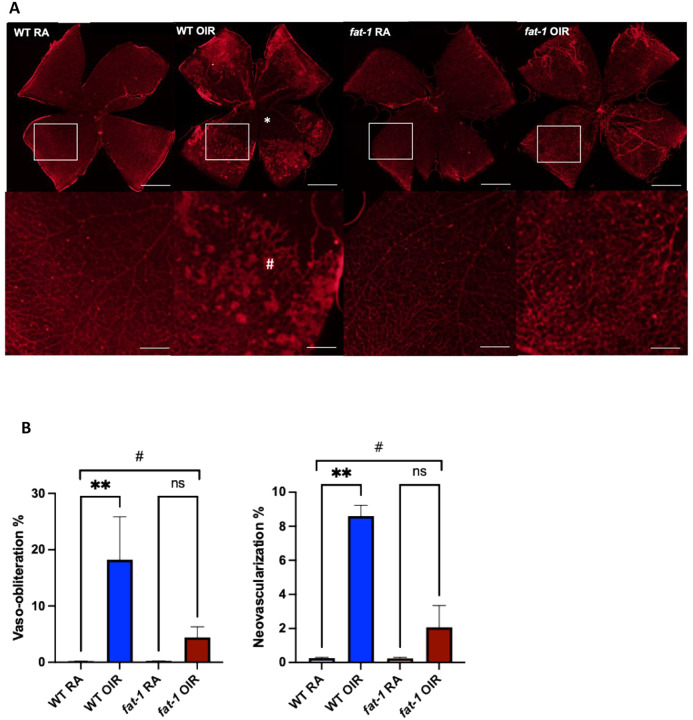
OIR-induced VO and NV and P17 is attenuated in *fat-1* mice compared to WT mice. (A) Retinal whole mount images of WT RA, WT OIR, *fat-1* RA, and *fat-1* OIR mice at P17. WT OIR demonstrated increased central VO* and NV^#^ compared to WT RA mice, while *Fat-1* OIR mice demonstrated no change in VO or NV compared to *fat-1* RA mice. Retinal whole mount images shown (top row), with endothelial staining (red) by immunofluorescence. Scale bars 500 μm. Enlarged inset (white box) images below. Scale bars on inset images = 125 μm. (B) Quantification of VO percentage and NV area percentage represented as median with IQR (n=5/group). # p≤ 0.0001by Kruskal-Wallis, **p≤0.01 by Dunn’s multiple comparisons test.

The *fat-1* transgenic mouse is a common model used to understand fatty acid metabolism.^24^ This genetic model overexpresses the *C. elegans fat-1* gene which converts ω−6 PUFAs to ω−3 PUFAs, resulting in elevated tissue levels of ω−3-PUFAs. Initially, we validated that *fat-1* mice were protected from pathological neovascularization. Pregnant WT and *fat-1* mice were subjected to hyperoxia at P7 for five days or maintained at room air. It has previously been shown that the degree of vaso-obliteration at P12 is similar between WT and *fat-1* mice.^21^ Whole mount imaging at P17 was then used to monitor the remaining degree of vaso-obliteration and corresponding pathological retinal neovascularization. As expected, whole mount imaging of WT RA and *fat-1* RA mice at P17 demonstrated normal retinal vascularization. However, WT OIR mice demonstrated significantly increased central vaso-obliteration at P17 compared to WT RA mice (WT RA 0.2% vs. WT OIR 18.0%, p=0.003) and concordant increase in peripheral pathological neovascularization (WT RA 0.3% vs. WT OIR 8.6%, p=0.009). In contrast, *fat-1* OIR mice were protected against the development of retinopathy, demonstrating no differences in vaso-obliteration (*fat-1* RA 0.3% vs *fat-1* OIR 4.4%, p>0.99) or neovascularization (*fat-1* RA 0.2 % vs. *fat-1* OIR 2.1%, p=0.5) compared to *fat-1* RA (Figure 3). These experiments confirm previous studies that the *fat-1* mice show attenuated vaso-obliteration and corresponding pathological neovascularization at P17 in OIR.^21^

### Differential transcriptional responses to OIR in WT and *fat-1* mice.

To understand globally how oxygen-induced vascularization is altered by ω−6:ω−3, we utilized whole retinal tissue RNA sequencing from WT and *fat-1* mice in RA and OIR conditions. To rule out the effect of differential cell-type composition in gene expression estimates, deconvolution analysis with reference cell types from the mouse body atlas^29^ was performed to confirm that all samples from WT and *fat-1* mice were maximally enriched in retina-specific expression, with marginal contribution from other cell type or tissue signatures (Supplemental Figure 1). In general, we did not detect significant baseline differences in the expression of lineage-defining genes for most retinal cell types^40^ between WT and *fat-1* mice, suggesting similar cellular compositions (Supplemental Figure 2).

**Figure 4:**
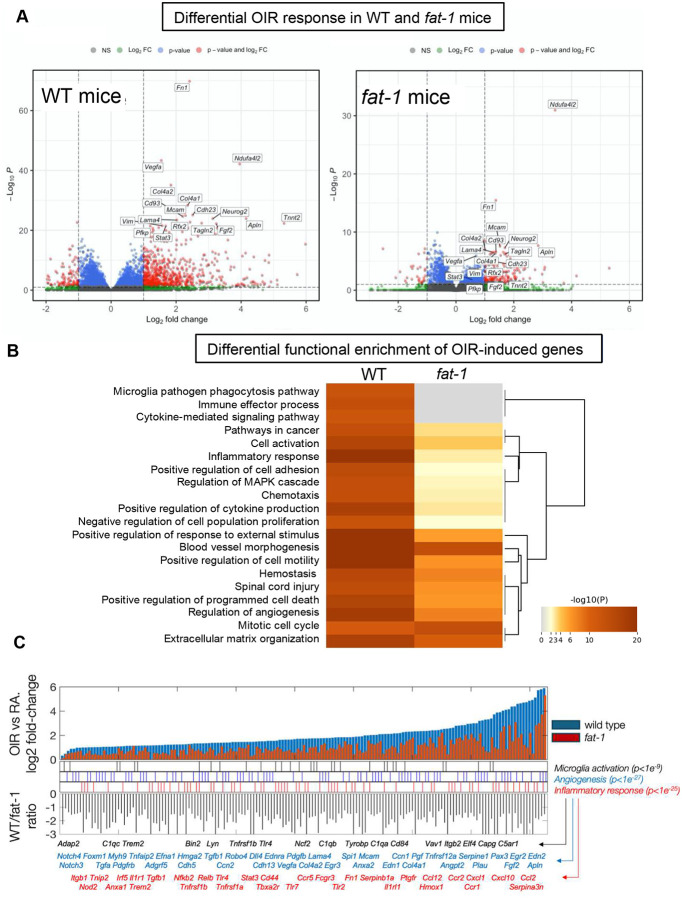
Differential pathway gene expression in WT and *fat-1* mice in response to OIR at P17. (A) Volcano plots of global response to OIR in WT (left) and *fat-1* mice (right). Highlighted are genes with maximal response in WT mice, with dampened or not significant response in *fat-1* mice. The x-axis represents log2 fold changes between OIR and RA mice and has the same scale in both plots. The y-axis represents -log10 adjusted p-values. Note the scale is different for both genotypes given the more marked statistical significance in WT mice. (B) Functional enrichment results of whole retina RNA sequencing. Significantly regulated genes in response to OIR as compared to RA were strongly enriched in inflammation, angiogenesis, and microglial activation pathways in wild type (WT) mice (left column in heatmap). These same pathways are less significant in *fat-1* mice (right column). (C) Differential regulation of key pathway genes in response to OIR in WT and *fat-1* mice. Shown are results for genes that exhibited strong up-regulation in WT mice and dampened or no up-regulation in *fat-1* mice in response to OIR. Top: bar plot of log2 fold changes (y axis) in OIR conditions as compared to RA for P17 mice in both genotypes. Genes are sorted by increasing fold change in WT mice (x axis). Middle: position of genes annotated in pathways significantly associated with OIR upregulation. Selected gene names are shown below. Bottom: bar plot of WT vs. *fat-1* log2 fold-change ratios.

However, we observed major differences in the global response to OIR between genotypes. Differential expression revealed a marked response to OIR in WT, with a dampened or less significant effect of OIR in *fat-1* mice (Figure 4A). Functional enrichment analysis demonstrated a distinct impact of OIR in inflammatory, angiogenic, and microglial activation pathways (Figure 4B), While we noted a distinct functional enrichment for genes with strong OIR-induced up-regulation in WT mice, we also observed a moderate or absent response to OIR in *fat-1* mice in pathways involving inflammatory response (adjusted p-value<10^−25^), angiogenesis (adjusted p-value<10^−27^), and microglial activation (adjusted p-value<10^−9^). (Figure 4C). These results suggest that the retinal ω−6:ω−3 modulates the transcriptional response to OIR, particularly in pathways know to mediate hyperoxic injury.

### *Fat-1* mice have attenuated OIR-induced microglial recruitment and activation and differential microglia subset enhancement compared to WT mice

Microglial activation and immune processes were among the top differentially altered pathways in WT mice but less impacted in *fat-1* mice (Figure 4). Within the central nervous system (CNS) and the retina, microglia serve as the primary resident immune cells. Although traditionally considered immune-privileged, microglia, a subset of myeloid cells, play a crucial role in regulating and maintaining the microenvironment in response to injury, hypoxia, neuroinflammation, degeneration, or aging. It has been shown that in response to these stimuli, microglia can undergo distinct activation processes including rapid proliferation, migration to affected areas, phagocytosis of cellular debris, and the release of cytokines, chemokines, or proangiogenic factors that influence neighboring retinal cells. Such interactions can contribute to pathological retinal neovascularization.^41–45^

**Figure 5:**
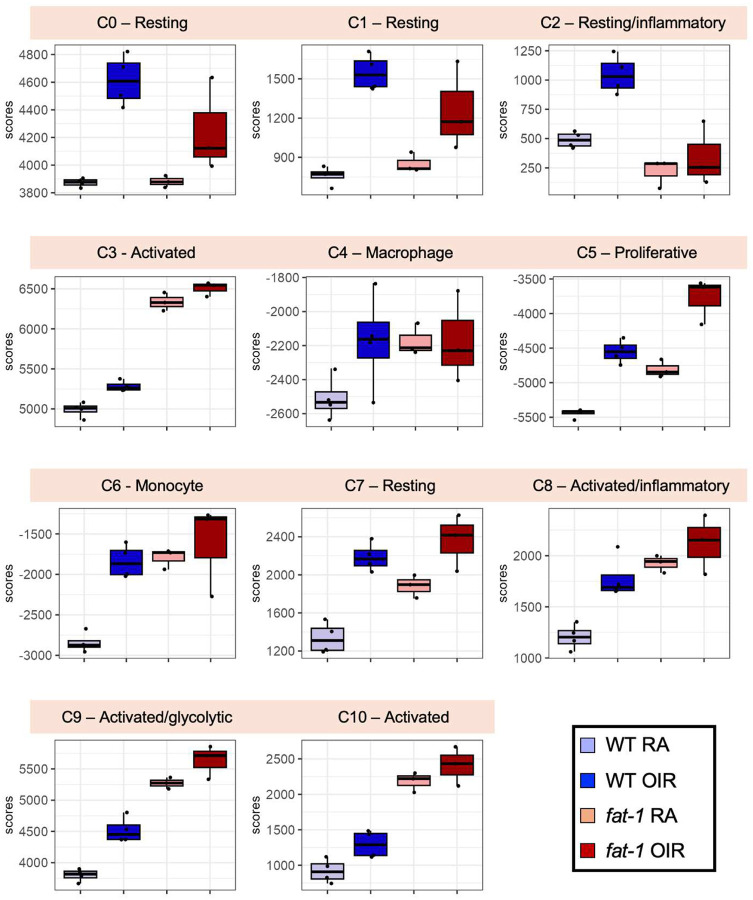
Microglial enrichment scores in WT and *fat-1* mice in RA and OIR conditions. Enrichment scores of WT and *fat-1* bulk RNA-Seq samples on a panel of microglia subtype signatures derived from single-cell profiling by Liu et. al. Cluster numbers (C0 through C10) corresponding to microglia subtypes and their phenotypic annotations are as defined in the publication.

We therefore performed enrichment analyses using fine-grained microglia signatures collected from high-resolution single-cell studies^25,32–35^ to understand if WT and *fat-1* mice are associated with distinct microglial activation phenotypes (Figure 5). In particular, using marker genes of resting and activated microglia subtypes in WT P17 RA and OIR mice defined by Liu *et. al*^25^, we observed that OIR induced an enrichment in proliferative phenotypes in both WT and *fat-1* mice (microglia subtype 5, Figure 5). Generic monocyte and resting microglia subsets (microglia subtypes 0, 1, 2, 4, 6, and 7) had significantly higher induction by OIR in WT mice, compared to *fat-1* mice. Higher enrichment in response to OIR for subtypes 2 and 8, representing pro-inflammatory resting and activated microglia, was predominantly observed in WT mice. Interestingly, *fat-1* mice demonstrated baseline enrichment compared to WT mice in precursor and activated microglial subtypes (subtypes 3, 5, 9 and 10) in both RA and OIR conditions. These subtypes were described by Liu *et. al*^25^ as intermediate and precursor microglia states that can differentiate into different functional subtypes in response to external insults, thus driving a reversible metabolic activation after OIR. Enrichment of these distinct subsets in *fat-1* mice suggest that a lower ω−6:ω−3 confers a higher phenotypic plasticity in response to OIR. Of note, compared to WT mice, *fat-1* mice show an attenuated response in genes associated with glycolytic microglia (Supplemental Figure 3A), with an attenuated response to OIR in *Slc16a3, Mt1, Gpi1*, and *Aldoa* genes. Microglia shift from oxidative phosphorylation towards glycolysis in inflammatory conditions such as diabetic retinopathy, producing acetyl-CoA which can modify gene expression towards pro-inflammatory responses. This glycolytic shift is encompassed within a larger microglial metabolic reprogramming which has been implicated in neurodegenerative disorders such as Alzheimer’s disease, some of which also affect the retina.^46^ In addition, WT mice showed clear upregulation of injury-related microglia genes^44^ in response to OIR (notably *Mrc1, Ccr1, Ms4a6c, Ms4a6b, Ms4a6d, Tmem176a*) indicative of a robust microglial injury response not observed in *fat-1* mice (Supplemental Figure 3B).

**Figure 6:**
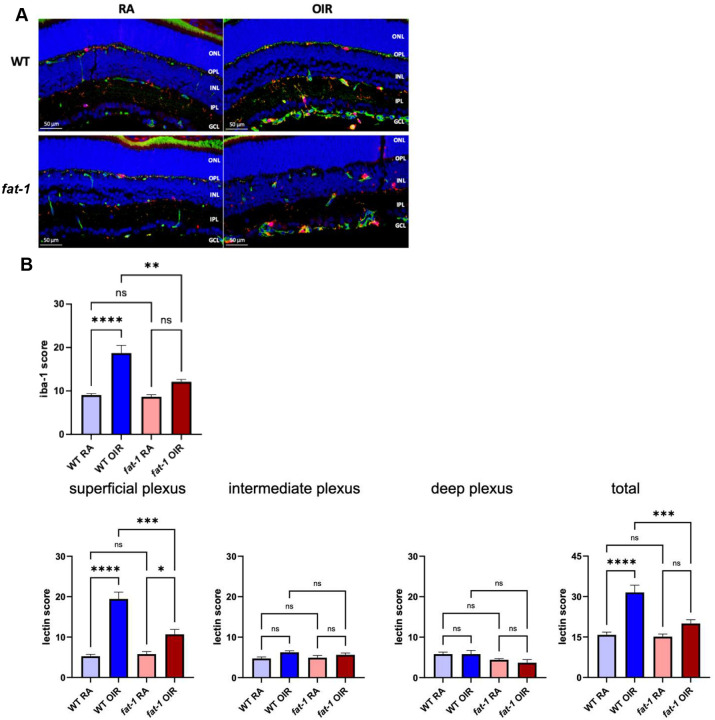
Microglia in WT and *fat-1* murine retina at P17 in RA and OIR conditions. (A) Detection of microglia in murine retina samples at P17 with anti-iba1 antibody (red) by immunofluorescence. Blood vessels indicated by anti-lectin antibody (green). ONL, outer nuclear layer; OPL, outer plexiform layer; INL, inner nuclear layer; IPL, inner plexiform layer; GCL, ganglion cell layer. Scale bars=50 μm. (B) Quantification of staining for iba-1 and lectin. Bars represent mean (SEM) in the amount of scoring. ****: p<0.0001; ***: p<0.001; **: p=0.0010, *: p=0.0289, by ANOVA one-way test followed by Bonferroni’s multiple comparisons test.

Given that RNA sequencing data overall demonstrated dampened microglial activation pathways in *fat-1* mice when exposed to OIR compared to WT mice exposed to OIR and that microglia are known to regulate local inflammation in the retina, we sought to characterize microglia *in situ*^47^. The baseline Iba1 microglial scoring at P17 in *fat-1* RA mice was similar to WT RA mice (WT RA 9.1±0.3 vs. *fat-1* RA 8.7±0.5, n=6–7/group; p>0.05). WT OIR mice demonstrated an increase in microglial Iba1 score compared to WT RA mice, particularly near the superficial vascular plexus, (WT RA 9.1±0.3; WT OIR 18.7±1.8; n=6/group; p<0.0001), whereas the *fat-1* OIR mice showed no significant differences in their microglial Iba1 score compared to *fat-1* RA mice (*fat-1* RA 8.7±0.5 vs. *fat-1* OIR 12.1±0.6; n=5–6/group; p>0.05) (Figure 6). As expected, the vascular staining was increased in the WT OIR group compared to the WT RA group in the superficial vascular plexus, but not in the *Fat-1* OIR group compared to *Fat-1* RA (WT RA 5.3±0.5 vs. WT OIR 19.5±1.7; n=6/group; p<0.0001). Lastly, we observed that the microglia largely co-localized along the retinal vascular plexuses.

### *Fat-1* mice exhibit dampened upregulation of ARA-derived oxylipins and DHA-derived resolvins in response to OIR, compared to WT mice

**Figure 7:**
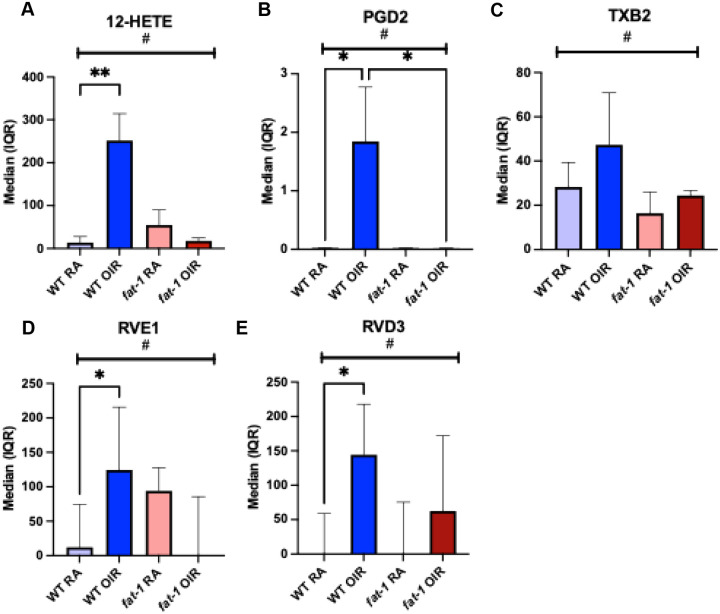
Retinal oxylipins and SPMs at P17 in WT and *fat-1* mouse retinas in RA and OIR conditions. Elevated concentrations (pg/mg) of (A) 12-Hydroxyeicosatetraenoic acid (HETE) (B) ProstaglandinD2 (PGD2) and (C) Thromboxane B2 (TXB2) (though this pairwise comparison did not reach significance for TXB2) in WT OIR mice compared to WT RA mice. *Fat-1* mice had decreased or stable concentrations of 12-HETE, PGD2, and TXB2 when exposed to OIR, compared to RA. Elevated concentrations of SPMs (D) RVE1 and (E) RVD3 in WT OIR when compared to WT RA. *Fat-1* OIR RVE1 and RVD3 concentrations were unchanged compared to *Fat-1* RA. ^#^P<0.05 when comparing all four groups by Kruskal-Wallis for all shown. *p<0.05 and **p≤ 0.01 analysis by Dunn’s multiple comparisons test.

Recent studies have shown a metabolic connection between the activation of microglia/macrophages and the stimulation of endothelial cells within the retinal angiogenic niche.^25^ To understand PUFA-mediated mechanisms that may shift the inflammatory microenvironment within the neuroretina, we quantified downstream bioactive lipid molecules at P17 in the setting of OIR. Three ARA-derived oxylipins, 12-hydroxyeicosatetraenoic acid (12-HETE), prostaglandin D2 (PGD2), and thromboxane B2 (TXB2) demonstrated a pattern of upregulation in WT OIR compared to WT RA, but no upregulation in *fat-1* OIR mice compared to *fat-1* RA. These oxylipins were significantly different when all four groups were compared (p< 0.02, by Kruskal-Wallis for all). In WT mice, 12-HETE was increased in conditions of OIR when compared to RA (WT RA 0.02 pg/mg, IQR: 0.01–20.92 vs. WT OIR 252 pg/mg, IQR: 82–314; p=0.002). In contrast, in *fat-1* OIR mice, there was no difference in 12-HETE compared to *fat-1* RA (*fat-1* RA 31.04 pg/mg, IQR: 0.03–86.87 vs. *fat-1* OIR 16 pg/mg, IQR: 5–32); p>0.99) (Figure 7A). PGD2 demonstrated a similar pattern. PGD2 was increased in WT OIR compared to WT RA and *fat-1* OIR (WT RA 0.018 pg/mg, IQR: 0.014–0.032 vs. WT OIR 1.12 pg/mg, IQR: 0.02–3.77; p=0.04) (WT OIR 1.12 pg/mg, IQR: 0.02–3.77 vs. *fat-1* OIR 0.019 pg/mg, IQR: 0.018–0.020; p=0.02) (Figure 7B).

Thromboxane B2 (TXB2), an inactive metabolite of thromboxane A2 (TXA2), was differentially expressed when four groups were compared, with the highest expression in WT OIR (WT RA 28 pg/mg, IQR: 15–39; WT OIR 47 pg/mg, IQR: 27–71; *fat-1* RA 16 pg/mg, IQR: 11–26; *fat-1* OIR 24 pg/mg, IQR: 12, 27; p=0.015) (Figure 7C).

Resolvin E1 (RVE1), a SPM derived from EPA, was increased in WT OIR when compared to WT RA (WT RA 12.09 pg/mg, IQR: 0.02–75.52; WT OIR 125 pg/mg, IQR: 88–216; p=0.03). There was a significant difference when comparing all groups (p=0.02 by Kruskal-Wallis) (Figure 7D). Likewise, resolvin D3 (RVD3), a DHA-derived SPM was increased in conditions of OIR when compared to RA in WT mice (WT RA 0.02 pg/mg, IQR: 0.01–59.08) vs. WT OIR 145 pg/mg, IQR: 80–218; p=0.02) (Figure 7E). In contrast, RVE1 and RVD3 concentrations in *fat-1* mice in OIR versus RA were not different, indicating an attenuated or no response in these resolvins in *fat-1* mice under OIR conditions. There was a significant difference when comparing all groups (p=0.02 by Kruskal-Wallis) (Figure 7D and 7E).

Other oxylipin and SPM measurements are shown in Supplemental Table 3.

## DISCUSSION

The pathophysiology of ROP is multifactorial. Extremely premature infants who develop chronic lung disease secondary to prolonged intubation and oxygen exposure are at high risk for severe ROP.^48^ Local hyperoxia induces inflammatory and oxidative cascades that dysregulate angiogenesis and immune responses, contributing to ROP.^49,^10 While supplementation of ARA and DHA in premature infants at risk for ROP appears to mitigate ROP outcomes,^9^ the optimal PUFA supplementation strategy and how ω−6:ω−3 protects against ROP is unknown.

Previous work has reported a decrease in ARA and an increase in retinal DHA in *fat-1* mice at P17 compared to WT mice in normoxic conditions.^21^ Here, we validated that *fat-1* OIR mice are protected against vaso-obliteration and neovascularization at P17, the *sine qua non* of OIR.^21^ Our study suggests that the protective effect of lower retinal ω−6:ω−3 is partially mediated by altered expression of ARA-derived oxylipins and ω−3-PUFA-derived SPMs with reduced activation of pro-inflammatory microglial subsets.

We utilized the *fat-1* mouse to evaluate how a decreased ω−6:ω−3 alters pathways involved in inflammation, angiogenesis, and immunity. As expected, OIR dramatically altered the retinal transcription of genes involved in these 3 pathways in WT mice, but this transcriptional shift in response to OIR was attenuated in *fat-1* mice. In response to OIR, *fat-1* mice showed weaker activation of several angiogenesis pathways relative to WT mice. The upregulation of *VEGFa*, the main isoform implicated in the pathologic angiogenesis of ROP, was dampened in *fat-1* mice compared to WT mice.^52^ This may have also protected *fat-1* OIR mice from the pathological retinal neovascularization that was observed in WT OIR mice. In fact, multiple angiogenic signaling genes were significantly more upregulated in WT OIR than in *fat-1* OIR (including *Pdgfb, Col4a2, Edn1, Ccn1, Angpt2, Socs3, Fgf2, Edn2, Aplnr, Sox9, Itgb1, Tgfa, Pdgfrb, Mmrn2, Elk3, Adgrf5, Robo4, Itga5, Ednra*). These genes have been implicated in retinal vascular development and other pathologic angiogenic eye disorders such as diabetic retinopathy, age-related macular degeneration, choroidal neovascularization, and ischemic retinopathy,^53–66^ underlying the strong association between ω−6:ω−3 and regulatory angiogenic networks.

An inflammatory milieu, spurred on by noxious factors such as infection or hyperoxia, has been shown to induce ROP.^67,68^ Our transcriptome analysis identified significant upregulation of many pro-inflammatory genes in WT OIR mice, but with a dampened response in *fat-1* OIR mice. Within the inflammatory pathway, proinflammatory genes such as N*fkb2, Tlr2, Tlr7, Cxcl1, Cxcl10, Ccl2*, and *Ccl12* were substantially increased in OIR vs. RA WT treated mice compared to *fat-1* mice exposed to the same conditions. *Cxcl1, Cxcl10, Ccl2* and *CCl12* have all been implicated in vascular permeability and immune recruitment in the context of retinal injury^69–72^. The overexpression of *Tlr2*, encoding a toll-like receptor that plays a role in pathogen recognition, could also be part of an innate response to OIR-induced retinal injury^73^. In agreement with our results, TLR2 has been reported to be inhibited by ω−3 PUFAs in macrophage cell culture^74,75^ and in animal models studying rosacea^76^.

In our study, nuclear factor kappa beta (NFKβ) and tumor necrosis factor (TNF) receptors were also significantly upregulated in WT OIR mice with little upregulation in *fat-1* OIR mice. Other studies corroborate this finding: in mice fed an ω−3-rich diet, there was suppression of TNFα, which is secreted from retinal microglia.^21^ Microglia are resident macrophages of the retina and regulate and influence retinal vascular development.^77^ Liu *et al*^78^ proposed that retinal microglia played a protective role in the retina; global retinal microglia inhibition via clondronate administration before hyperoxia exposure increased vaso-obliteration and neovascularization.

This highlights that differing microglial states, which can be altered by noxious stimuli in the local retinal environment, fuel important injury responses that can serve to worsen or mitigate retinopathy. The integration of our transcriptome sequencing data from the whole retina in WT and *fat-1* mice and the single-cell signatures reported by Liu *et al*^78^ lend support to these findings, demonstrating a shift to activated and proliferative microglia in response to OIR.

While both WT and *fat-1* mice demonstrate a shift to proliferative microglia in OIR conditions, WT mice demonstrate a more marked shift in certain “resting” microglial clusters to OIR conditions compared to *fat-1* mice. Interestingly, *fat-1* mice show a higher baseline enrichment in “activated/inflammatory” microglial signatures in RA, which may explain why these signatures experienced an enhanced or “catch up” response to OIR in WT mice. When we took a closer look at these microglial clusters, microglial injury and the glycolytic microglial response was dampened in *fat-1* mice in response to hyperoxia. This pattern suggests that a decreased ω−6:ω−3 attenuates OIR-induced microglial pro-inflammatory activation in certain microglial subsets. Interestingly in WT mice, this shift in response to OIR is accompanied by recruitment of microglia *in situ*, whereas *fat-1* mice do not demonstrate microglial recruitment. Our findings suggest that altered retinal PUFA balance mediates microglial activation via shifts in glycolysis. A shift to glycolysis from oxidative phosphorylation is a key metabolic alteration in inflammatory macrophages.^34^

Amongst the many potential effects that PUFA-derived SPMs could have on the neuroretinal microenvironment, it is already established that these SPMs affect microglial activation.^79^ Resolvins (resolution phase interaction products) are bioactive products derived from EPA and DHA^21^ that promote inflammatory resolution.^80,81^ They have been described to have a protective effect against diabetic retinopathy in animal models^82,83^ and aberrant hyperglycemia-induced angiogenic signaling *in vitro*^84^. In our study, in WT mice, hyperoxia generated a robust RVE1 and RVD3 response to possibly help curb retinal inflammation. We also observed an attenuated increase in RVD3 and a decrease in RVE1 in *fat-1* OIR mice compared to WT OIR mice. Connor et al^21^ demonstrated a similar pattern; retinal resolvin E2 (RVE2), a biosynthetic marker for RVE1 was decreased at P17 in WT mice fed an ω−3 rich diet after hyperoxia exposure compared to normoxia conditions.

In addition to the changes observed in ω−3 derived SPMs, we observed changes in ARA-derived oxylipins. Oxylipins are generated via oxygenation of PUFAs, suggesting that they may act as a sensor of aberrant oxygen exposure. 12-HETE and PGD2, two ARA-derived oxylipins, were increased in the WT OIR group vs. WT RA group. However, this response in 12-HETE and PGD2 was not observed in *fat-1* mice in response to OIR. 12-HETE is derived from ARA via 12-lipoxygenases (12-LOX).^85^ There is evidence that 12-LOX and its product, 12-HETE, play a role in regulating inflammatory and angiogenic processes that underlie diabetic retinopathy.^86^ 12-HETE has also been shown to increase VEGF expression in human and porcine vascular smooth cell cultures.^87^ In a human metabolomics study of diabetic retinopathy, serum 12-HETE was a better predictive marker for diabetic retinopathy than HgbA1c, a standard test for assessing glycemic control.^88^

PGD2 is generated via the enzyme cyclooxygenase; it is abundant in the choroid.^89^ In our study OIR increased PGD2. PGD2 has been shown to mediate choroid involution and endothelial cell apoptotic death induced by OIR.^90,91^ We speculate that hyperoxic injury induces ARA-derived oxylipins such as PGD2 that promote aberrant angiogenesis and inflammation, but a pro-resolution response is mounted by the metabolism of ω−3 PUFAs (RVD3, RVE1) in WT mice. A decreased retinal ω−6:ω−3 in the setting of OIR mediates attenuation of the hyperoxia-induced increase in ARA-derived oxylipins and reduced the extent of EPA and DHA-derived resolvin-mediated pro-resolution response.

Our study has limitations. Our study was not powered to detect potential sex differences, which are known to have an effect in lipid metabolism.^92^ Lastly, because *fat-1* mice skew ω−6:ω−3, we cannot disentangle the effect of individual PUFAs on the retinal phenotype.

In conclusion, our study demonstrates that a decreased *ω*−6:*ω*−3 protected against aberrant retinal vascularization induced by OIR. This protection may be mediated by complex interactions of PUFAs and generation of downstream bioactive lipid molecules that dampen inflammation, aberrant angiogenesis, and pathological microglial recruitment and activation after hyperoxia exposure. Further research is needed to precisely understand how PUFAs regulate retinal development and function and long-term vision in the preterm infant.

## Supplementary Material

1

## Data Availability

The raw RNA sequencing dataset generated and/or analyzed during the current study has been deposited in the National Center for Biotechnology Information (NCBI) Short Read Archive (SRA) with accession code PRJNA 1006021. Data from our previous study^26^ is available at NCBI’s Gene Expression Omnibus (GSE123945),

## ABBREVIATONS:

12-HETE: 12-hydroxyeicosatraenoic acid
ALA: α-linoleic acid
ARA: arachidonic acid
DGLA: dihomo-gamma-linolenic acid
DHA: docosahexaenoic acid
DPA: docosapentaenoic acid
DTA: docosatrienoic acid
EDA: eicosadienoic acid
EPA: eicosapentaenoic acid
ETE: eicosatrienoic acid
FAME: fatty acid methyl ester
GC-MS: gas chromatography-mass spectrometry
HDHA: hydroxy-docosahexaenoic acid
IF: immunofluorescence
LA: linoleic acid
LC-MS: liquid chromatography-mass spectrometry
MA: mead acid
OIR: oxygen-induced retinopathy
PGD2: prostaglandin D2
PPAR: peroxisome proliferator-activated receptor
PUFA: polyunsaturated fatty acid
RA: room air
ROP: retinopathy of prematurity
VEGF: vascular endothelial growth factor
WT: wild type
