# Supplementary material for: Polyunsaturated fatty acid balance modulates microglial state in a murine model of oxygen-induced neovascularization
Source: Res Sq. 2025 Sep 23:rs.3.rs-7491382. Preprint. [Version 1] doi: 10.21203/rs.3.rs-7491382/v1 (PMC12486074; doi:10.21203/rs.3.rs-7491382/v1)
Supplement: 1 [file NIHPPRS7491382V1-supplement-1.pdf]

Supplemental Table 1: Mouse Diets

	PicoLab Rodent Diet 20	Mod TestDiet 58B0 with 10% corn oil
**Fat %**	5	10
Linoleic Acid %	2.32	5.72
Linolenic Acid %	0.28	0.09
Arachidonic Acid %	0.02	0.00
O-3 Fatty acid, %	0.42	0.09
Total Saturated Fatty acids, %	0.77	1.27
Total Monounsaturated Fatty Acids, %	1.0	2.42
Cholesterol, ppm	135	0
**Protein, %**	21	21
Arginine, %	1.29	0.82
Histidine, %	0.53	0.61
Isoleucine, %	0.87	1.13
Leucine, %	1.58	2.03
Lysine, %	1.18	1.71
Methionine, %	0.62	0.96
Cystine, %	0.36	0.09
Phenylalanine, %	0.92	1.13
Tyrosine, %	0.61	1.19
Threonine, %	0.79	0.91
Tryptophan, %	0.24	0.26
Valine, %	0.97	1.34
Alanine, %	1.20	0.65
Aspartic Acid, %	2.23	1.52
Glutamic Acid, %	4.26	4.80
Glycine, %	0.98	0.45
Proline, %	1.32	2.77
Serine, %	1.00	1.30
Taurine, %	0.03	0.00
**Carbohydrates, %**	53.5	56.3
Fiber, %	4.4	5.9
**Calories Provided by**		
Fat, %	13.1	22.5
Protein, %	24.5	21.0
Carbohydrates, %	62.4	56.4

Supplemental Table 2: Oxylipin analysis parameters

Oxylipin		Precursor Mass (M1)	Product Mass (M2)	Retention Time (min)	LOD (pg/µl)	LLOQ (pg/µl)
Common name	Abbrev.					
9-Hydroxyoctadecadienoic acid	9-HODE	295.2	171.1	16.00	0.042	0.042
13-Hydroxyoctadecadienoic acid	13-HODE	295.2	195.1	15.81	0.064	0.064
6-keto Prostaglandin F1α	6k-$PGF1_{\alpha}$	369.2	163.1	7.40	0.004	0.004
Thromboxane B2	TXB_2	369.2	169.1	8.26	0.009	0.009
Prostaglandin F2α	$PGF_{2\alpha}$	353.2	193.1	9.64	0.019	0.019
Prostaglandin E2	PGE_2	351.2	271.2	8.59	0.020	0.020
Prostaglandin D2	PGD_2	351.2	233.1	8.68	0.016	0.016
12-Hydroxyheptadecatrenoic acid	12(S)-HHTre	279.2	179.2	14.06	0.241	0.241
15-Hydroxyeicosatetraenoic acid	15-HETE	319.2	219.11	16.03	0.014	0.014
12-Hydroxyeicosatetraenoic acid	12-HETE	319.2	179.2	16.41	0.975	0.975
5-Hydroxyeicosatetraenoic acid	5-HETE	319.2	115.1	16.58	0.919	0.919
Lipoxin B_4	LXB_4	351.2	221.1	9.79	0.004	0.004
Lipoxin A_4	LXA_4	351.2	115.11	10.56	0.023	0.023
14,15-epoxyeicosatrienoic acid	14,15 EET	319.2	219.1	16.73	0.023	0.023
8,9-Epoxyeicosatrienoic acid	8,9 EET	319.2	155.1	17.45	1.805	2.276
15(R)-Lipoxin A_4	15(R)-LXA_4	351.2	115.1	10.57	0.444	0.444
Resolvin E_1	RvE_1	349.2	195.1	6.32	0.006	0.006
18-hydroxyicosapentaenoic acid	18-HEPE	317.2	259.1	15.10	0.003	0.003
5-Hydroxyeicosapentaenoic acid	5-HEPE	317.2	115.1	15.57	0.012	0.012
15-Hydroxyeicosapentaenoic acid	15-HEPE	317.2	219.1	15.44	0.018	0.018
Resolvin D_3	RvD_3	375.2	147.1	9.54	0.788	0.788
Resolvin D_2	RvD_2	375.2	175.1	9.43	0.917	0.917
17(R)-resolvin D_1	17(R)-RvD_1	375.2	141.1	10.54	0.022	0.022
Resolvin D_1	RvD_1	375.2	141.11	10.35	0.018	0.018
Resolvin D_5	RvD_5	359.2	199.1	13.03	0.108	0.108
4-hydroxydocosahexaenoic acid	4-HDHA	343.2	101.1	17.23	0.015	0.015
7-hydroxydocosahexaenoic acid	7-HDHA	343.2	141.1	16.66	0.066	0.066
13-hydroxydocosahexaenoic acid	13-HDHA	343.2	193.1	16.62	0.030	0.030
14-hydroxydocosahexaenoic acid	14-HDHA	343.2	205.1	16.46	0.003	0.003
17-hydroxydocosahexaenoic acid	17-HDHA	343.2	245.1	16.51	0.008	0.012
9S-HODE-d4		299.1	172.3			
6k-PGF1a-d4		373.4	167			
TXB2-d4		373	173			
PGF2a-d4		357.2	193.1			
PGE2-d4		355.2	193.1			
PGD2-d4		355.1	193.11			
LTB4-d4		339.2	197.1			
12-HETE-d8		327.2	184.1			
5-HETE-d8		327.2	116.1			
14,15 DiHETrE-d11		348.2	207.1			
RvD3-d5		380.2	152.1			
RvD2-d5		380.2	141.1			

Supplemental Table 3: Retinal oxylipins and the PUFA responsible for generating the oxylipin of interest at P17.

Oxylipin	9 HODE	13 HODE	TXB2	PGF2a	PGE2	PGD2	15-HETE
**PUFA**	LA	LA	ARA	ARA	ARA	ARA	ARA
**WT RA (n=6)**	16 (7, 32)	13 (5, 29)	28 (15, 39)	55 (30, 88)	32 (25, 58)	0.18 (0.014, 0.032)	174 (93, 186)
**WT OIR (n=6)**	12 (5, 30)	6 (2, 19)	47 (27, 71)	29 (16, 45)	12 (6, 38)	1.1‡ (0.02, 3.8)	202 (109, 267)
***fat-1* RA (n=6)**	32 (28,44)	21 (18, 37)	16 (11, 26)	35 (0.03, 48)	40 (36, 66)	0.023 (0.022, 0.029)	50 (14, 182)
***fat-1* OIR (n=5)**	70 (28,176)	46# (20,138)	24 (12, 27)	0.02 (0.02, 29)	44 (33, 51)	0.019# (0.018, 0.020)	16 (11, 455)
**p value**	0.03	0.03	0.02	0.1	0.09	0.01	0.2

**Oxylipin**	12-HETE	5-HETE	LXB4	LXA4	14-15 EET	8 9 EET	15 R LXA4
**PUFA**	ARA	ARA	ARA	ARA	ARA	ARA	ARA
**WT RA (n=6)**	0.02 (0.01, 20.9)	108 (0.02, 718)	1674 (865, 5099)	425 (164, 2026)	0.02 (0.01, 0.03)	667 (0.02, 2215)	427 (159, 1831)
**WT OIR (n=6)**	252 ‡ (82,314)	228 (192, 347)	1050 (623, 2897)	184 (119, 962)	0.03 (0.03, 118)	0.03 (0.03, 1533)	189 (158,938)
***fat-1* RA (n=6)**	31 (0.03, 87)	212 (10, 413)	2874 (1538, 4246)	661 (540, 1201)	0.02 (0.02, 0.03)	0.03 (0.02, 124)	657 (552, 1201)
***fat-1* OIR (n=5)**	16 (5, 32)	305 (154. 520)	2077 (1099, 2610)	510 (227, 663)	0.02 (0.02, 128)	0.02 (0.02, 3028)	503 (85, 633)
**p value**	0.004	0.7	0.5	0.1	0.09	0.6	0.1

**Oxylipin**	RVE1	18 HEPE	5 HEPE	15 HEPE	RVD3	RVD2	17 R RV
**PUFA**	EPA	EPA	EPA	EPA	DHA	DHA	DHA
**WT RA (n=6)**	12 (0.02, 75)	37 (26, 112)	129 (34, 413)	392 (234, 494)	0.02 (0.01, 59)	0.017 (0.02, 372)	0.02 (0.02, 26)
**WT OIR (n=6)**	125‡ (88, 216)	7903 (42, 63158)	461 (138, 780)	817 (223, 1494)	145‡ (80, 218)	0.03 (0.02, 166)	25 (8, 80)
fat-1* RA (n=6*)	94 (0.03, 128)	154 (67, 401)	288 (227, 538)	323 (135, 1116)	0.03 (0.02, 76)	0.02 (0.02, 75)	24 (19, 28)
***fat-1* OIR (n=5)**	0.02 (0.02, 86)	72 (18, 289)	162 (57, 313)	62 (45, 1185)	63 (27, 172)	0.02 (0.02, 0.02)	23 (0.02, 181)
**p value**	0.02	0.2	0.2	0.4	0.02	0.05	0.2

**Oxylipin**	RVD1	RVD5	4 HDHA	7 HDHA	13HDHA	14 HDHA	17 HDHA
**PUFA**	DHA	DHA	DHA	DHA	DHA	DHA	DHA
**WT RA (n=6)**	0.02 (0.01, 18)	0.02 (0.02, 26)	0.02 (0.01, 14)	404 (135, 688)	37 (19, 57)	84 (54, 153)	357 (87, 621)
**WT OIR (n=6)**	39 (0.03, 59)	0.03 (0.02, 4)	0.03 (0.02, 102)	672 (354, 1423)	134 (76, 210)	302 (176, 325)	657 (456, 1556)
***fat-1* RA (n=6)**	7 (0.03, 29)	4 (0.02, 8)	0.02 (0.02, 0.03)	566 (277, 690)	38 (29, 136)	111 (45, 182)	209 (20,712)
***fat-1* OIR (n=5)**	0.02 (0.02, 91)	12 (0.02, 53)	0.02 (0.02, 123)	634 (446, 2187)	36 (11, 252)	51 (23, 381)	146 (92, 1999)
**p value**	0.08	0.6	0.1	0.3	0.3	0.09	0.2

Data represented as median (25th percentile, 75th percentile). P value analysis between all four groups by Kruskal-Wallis. In order to assess the potential for false discoveries, we computed the False Discovery Rate (FDR) using the Benjamini/Hochberg method.[68] A p-value threshold of 0.05 corresponded to an FDR rate of 17.5%. This indicates that of the seven variables found to

be significant, approximately 17.5% (or approximately 1.2) could be false positive findings. Pair-wise comparisons by Dunn's multiple comparisons test as follows:

‡ $p<0.05$ between WT RA and WT OIR

# $p<0.05$ between WT OIR vs. *fat-1* OIR

HODE, Hydroxyoctadecadienoic acid; TXB, thromboxane; PG, prostaglandin; HETE, hydroxyeicosatetraenoic acid; LX, lipoxin; EET, epoxyeicosatrienoic acid; RV, resolvin; HEPE, hydroxyeicosapentaenoic acid; HDHA, hydroxy-docosahexaenoic acid

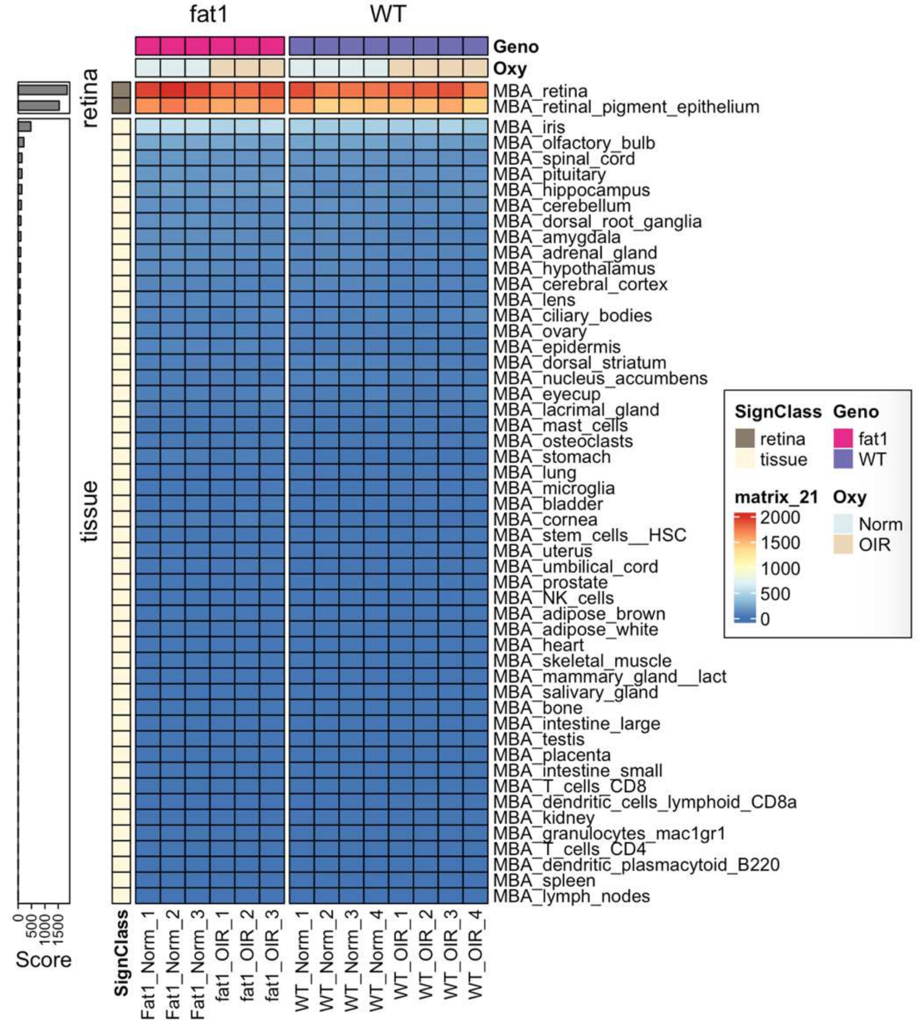

Supplementary Figure 1: A: Deconvolution analysis of whole-retina RNA-Seq samples from this study. Samples are grouped according to genotype (*Geno)* and oxic condition (Oxy). All signatures from the mouse body atlas (MBA) were used as a reference for enrichment analysis. The heatmap represents the raw enrichment scores of each sample across all reference tissues, which are sorted in decreasing order of average enrichment across the dataset (left barplot). The two MBA retinal signatures showed the highest enrichment score for all samples, with marginal representation of non-retinal signatures. **B**: Boxplot of deconvolution scores for the microglial signature. Enrichment scores in OIR samples are significantly higher than in RA samples (Kolmogorov-Smirnov test p-value$<5x10^{-4}$).

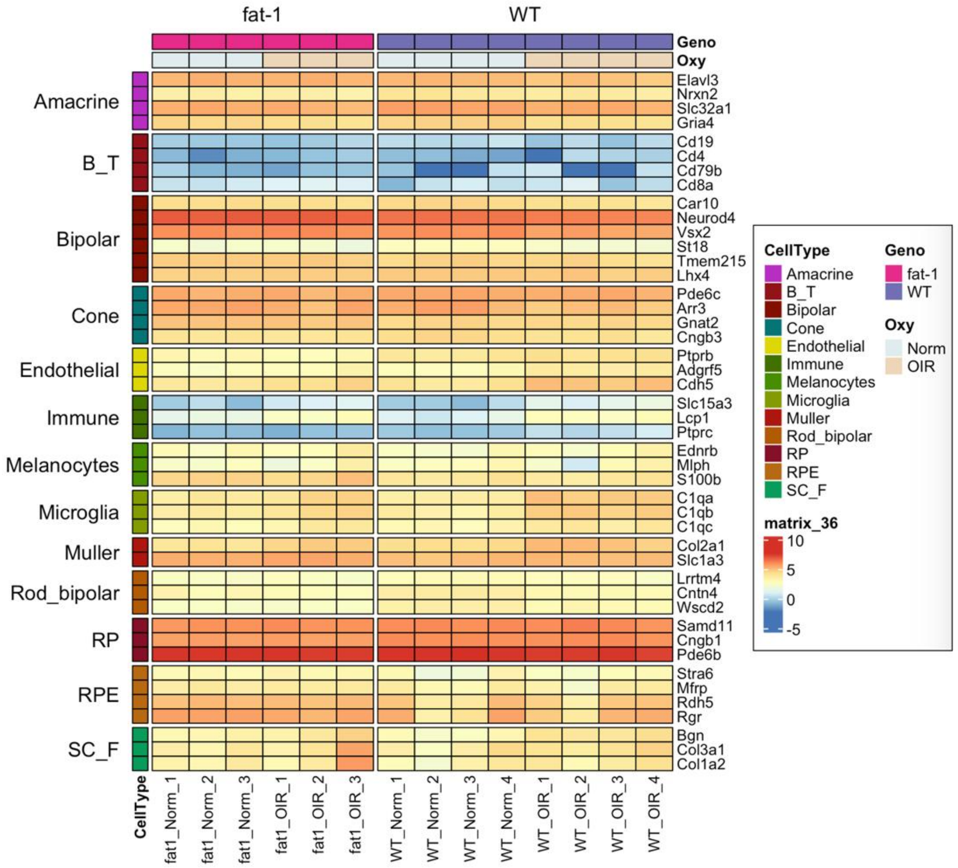

Supplementary Figure 2: Gene expression heatmap of cell-type markers for retinal cell population curated from previous studies. The heatmap represents log-normalized expression estimates for all samples in this study (colormap). Samples are grouped according to genotype (*Geno)* and oxic condition (Oxy). Genes are grouped according to the cell-type they represent (left annotations). *B_T (B or T cells), RP=rod photoreceptors, RPE=retinal pigment epithelium; SC_F= Sclera & Cornea and Fibroblasts; Muller=Müller Glial cells; Cone=Cone photoreceptors; Bipolar=Bipolar cells.*

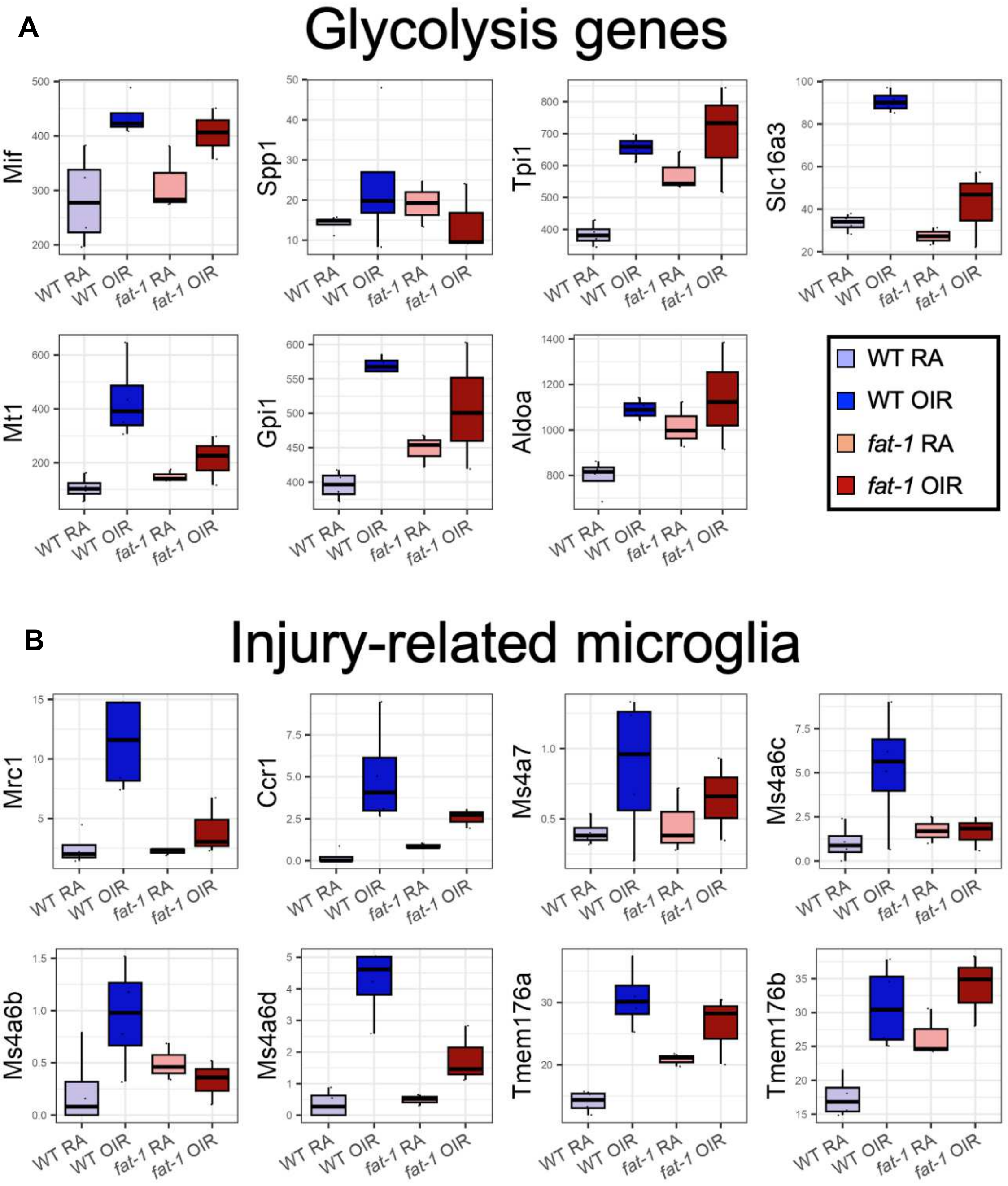

Supplementary Figure 3: (A) Gene expression of glycolytic microglia marker genes in WT and *fat-1* mice under RA and OIR conditions. Genes were retrieved from single-cell studies characterizing murine microglia subtypes (Liu, Z. *et al.* and Hammond, T. R. *et al.*) (B) Bulk RNA-Seq gene expression of injury-related microglia genes (Hammond, T. R. *et al*) in WT and *fat-1* mice under RA and OIR conditions.
